# Infection of sheep by *Echinococcus multilocularis* in Gansu, China: evidence from mitochondrial and nuclear DNA analysis

**DOI:** 10.1186/s40249-023-01120-0

**Published:** 2023-08-10

**Authors:** Nigus Abebe Shumuye, Li Li, Wen-Hui Li, Nian-Zhang Zhang, Yan-Tao Wu, Yao-Dong Wu, Wen-Jun Tian, Lin-Sheng Zhang, Xiao-Feng Nian, Guo-Dong Dai, Wei-Gang Chen, Sheng-Zhi Gao, Xue-Qi Tian, Jun-Shi Liu, Bin Li, Nigatu Kebede, Bao-Quan Fu, Hong-Bin Yan, Wan-Zhong Jia

**Affiliations:** 1 State Key Laboratory for Animal Disease Control and Prevention, College of Veterinary Medicine, National Para-Reference Laboratory for Animal Echinococcosis, Key Laboratory of Veterinary Parasitology of Gansu Province Lanzhou Veterinary Research Institute, Lanzhou University, Chinese Academy of Agricultural Sciences, Lanzhou, 730046 China; 2 Department of Veterinary Clinical Medicine and Epidemiology, Mekelle University, College of Veterinary Sciences, Kalamino Campus, P.O.Box: 2084, Mekelle, Tigray Ethiopia; 3 Gansu Animal Centre for Disease Control and Prevention, Lanzhou, 730046 Gansu Province China; 4 Jingyuan County Animal Centre for Disease Control and Prevention, Jingyuan County Animal Husbandry and Veterinary Technical Service Center, Jingyuan, 730600 Gansu Province China; 5 Aklilu Lemma Institute of Pathobiology, Addis Ababa University, Addis Ababa, Ethiopia; 6 Jiangsu Co-Innovation Center for Prevention and Control of Important Animal Infectious Diseases and Zoonoses, Yangzhou, 225009 China

**Keywords:** *Echinococcus multilocularis*, China, Sheep, Liver, Phylogeny

## Abstract

**Background:**

In the normal life cycle of the parasite (*Echinococcus multilocularis*) that causes alveolar echinococcosis, domestic and wild carnivores act as definitive hosts, and rodents act as intermediate hosts. The presented study contributes to the research on the distribution and transmission pattern of *E. multilocularis* in China having identified sheep as an unusual intermediate host taking part in the domestic transmission of alveolar echinococcosis in Gansu Province, China.

**Methods:**

From 2020 to 2021, nine whitish different cyst-like were collected from the liver of sheep in Gansu Province for examination. A near complete mitochondrial (*mt*) genome and selected nuclear genes were amplified from the cyst-like lesion for identification. To confirm the status of the specimen, comparative analysis with reference sequences, phylogenetic analysis, and network analysis were performed.

**Results:**

The isolates displayed ≥ 98.87% similarity to *E*. *multilocularis* NADH dehydrogenase sub-unit 1 (*nad*1) (894 bp) reference sequences deposited in GenBank. Furthermore, amplification of the *nad*4 and *nad*2 genes also confirmed all nine samples as *E*. *multilocularis* with > 99.30% similarity. Additionally, three nuclear genes, pepck (1545 bp), elp-exons VII and VIII (566 bp), and elp-exon IX (256 bp), were successfully amplified and sequenced for one of the isolates with 98.42% similarity, confirming the isolates were correctly identified as *E. multilocularis*. Network analysis also correctly placed the isolates with other *E. multilocularis*.

**Conclusions:**

As a result of the discovery of *E. multilocularis* in an unusual intermediate host, which is considered to have the highest zoonotic potential, the result clearly demonstrated the necessity for expanded surveillance in the area.

**Graphical Abstract:**

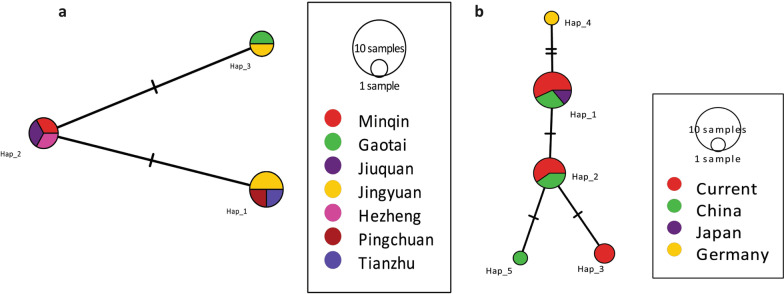

**Supplementary Information:**

The online version contains supplementary material available at 10.1186/s40249-023-01120-0.

## Background

In the well-known wildlife transmission cycle of *Echinococcus multilocularis*, rodents and other small mammals (e.g., *Microtus* and *Alticola* voles; water voles, *Arvicola* spp.; shrews; muskrats, *Ondatra zibethicus*; nutria; *Myocastor coypus*) serve as intermediate hosts, while foxes such as red foxes (*Vulpes vulpes*), Arctic foxes (*Alopex lagopus*), Tibetan foxes (*V. ferrilata*) and sand foxes (*V. corsac*) serve as definitive hosts [1, 2]. In certain areas (such as sections of Alaska and China), a semidomestic or synanthropic cycle with domestic carnivores (dogs) as the definitive host has been demonstrated [3]. In the intermediate host, *E. multilocularis* metacestodes produce a multilocular (multichambered) mass that contains hundreds to thousands of protoscolices in some and fewer to none in others [4].

In many countries, the distribution of *E*. *multilocularis* is not uniform, with areas with high and low prevalence zones. These disparities have been linked to the use and structure of the landscape (vegetation type, vegetation covering, grassland ratio, and elevation), as well as climatic elements (temperature and humidity), which determine the species range and abundance of rodent intermediate hosts [5]. More so, there is mounting evidence that *E*. *multilocularis* is expanding both its geographic and host range as a result of anthropogenic influences and has been linked to changes in the distribution of definitive hosts [6–8]. Depending on the location, *E*. *multilocularis* tapeworms can be found in 1–60% of wild canids (particularly foxes) and less than 1% of dogs and cats. In China, up to 12% of dogs can be infected with *E. multilocularis*, but the cysts are commonly found in less than 0.5% to 10% of livestock [9].

Generally, both *E*. *granulosus* sensu lato and *E*. *multilocularis* have multi-host systems that are influenced by environmental factors such as the number of host species, habitats, density, predator–prey relationships, and egg survival [10–16].

Alveolar echinococcosis (AE) is common in the northern hemisphere, including western, northern, and eastern Europe, Russia, western Alaska, the northwestern portion of Canada, central Asia, northern Japan, and western and northern China [17–21]. In China, AE is endemic in 99 counties across six provincial-level administrative divisions, including Qinghai, Sichuan, Gansu, Tibet, Xinjiang, and Ningxia. Most of these counties are concentrated in the Qinghai-Tibet Plateau, especially in the border area between Qinghai and Sichuan provinces, as well as in the Tibet Autonomous Region, where 13 of the 15 counties with the highest prevalence of AE have been recorded [22].

Genetic characterization using mitochondrial deoxyribonucleic acid (*mt*DNA) markers, the microsatellite EmsB, and a multi-locus nuclear DNA markers were applied to *E. multilocularis* genotyping studies [23]. Although *E. multilocularis* is less diverse than *E. granulosus* sensu lato, recent taxonomic studies have shown intra-specific variations within *E. multilocularis* at the global scale, allowing the identification of four genetic subgroups that are restricted to specific geographic regions: European, North American, Asian, and Mongolian [8, 24–26].

When a suspected or confirmed case of *E. multilocularis* infection is reported in any domestic animal, public health agencies must assess the risk of transmission to humans. Such proactive measures can help halt the spread of *E. multilocularis* [27]. The aim of the current study was the molecular characterization, including phylogenetic analysis, genetic diversity, and network analysis, of *E. multilocularis* detected in sheep in Gansu, China.

## Methods

### Study area

Gansu is a landlocked province in the northwest of China located at 32°31′–42°57′N and 92°13′–108°46′E with a semi-arid to arid continental climate. There are fourteen administrative divisions in Gansu: 12 prefecture-level cities and two autonomous prefectures which are subdivided into 86 county-level divisions [28]. Samples were collected by the Gansu Provincial Center for Animal Disease Control and Prevention from different counties in different prefecture-level cities: Minqin and Tianzhu counties (Wuwei City), Jingyuan and Pingchuan counties (Baiyin City), Hezheng county (Linxia City), Gaotai county (Zhangye City), and Jiuquan City. The geographic coordinates of the survey sites are shown in Table 1, and the study area is presented in Additional file 1: Fig. S1.

**Table 1:** Geographic coordinates of the survey sites

Prefecture-level city	County	Latitude	Longitude
Wuwei	Minqin	38°37′39.36′′	103°5′32.2152′′
	Tianzhu	37°08′23.84′′	102°46′23.09′′
Zhangye	Gaotai	39°22′09′′	99°48′49′′
Baiyin	Jingyuan	36°34′48′′	104°36′36′′
	Pingchuan	36°43′39′′	104°49′30′′
Linxia	Hezheng	35°25′29′′	103°21′04′′
Jiuquan		39°43′56.9568′′	98°29′39.8796′′

### Parasitic material, DNA extraction, PCR amplification and sequencing of mitochondrial and nuclear DNA

During a routine cystic echinococcosis (CE) investigation suspected (whitish lesion) samples of nine sheep from different slaughterhouses were collected. The infected livers were separated from the carcass. Then the cyst-like samples were separated from the infected liver and washed three times with phosphate buffered saline; thereafter, they were kept at – 20 ℃ until DNA extraction. Each sample was treated as a separate isolate. Following the manufacturer's instructions, total genomic DNA was extracted using the TIANamp Genomic DNA Kit (TIANGEN Biotech Co., Ltd., Beijing, China). PCR amplification and sequencing were achieved for the NADH dehydrogenase subunit 1 (*nad*1) mitochondrial (*mt*) gene (~ 1280 bp) using forward (5′-ATTATAGAAAATTTTCGTTTTACACGC-3′) and reverse (5′-ATTCACAATTTACTATATCAAAGTAACC-3′) primers [29]. This was proceeded by National Center for Biotechnology Information Basic Local Alignment Search Tool (NCBI BLAST, https://blast.ncbi.nlm.nih.gov/Blast.cgi) for identification. Thereafter, a near-complete *mt* genome (Table 2) and nuclear genes (Table 3) were amplified for confirmation obtained from previous published papers and newly designed primer pairs using Premier 5 software [30].

**Table 2:** Primers for PCR amplification for mitochondrial genes of *Echinococcus multilocularis* with positions based on a reference sequence (AB018440 in GenBank)

Gene	Primer name	Primer sequence (5′–3′)	Length	Position	Size (bp)	References
*nad*5	Emnd5-F	CTATTATGGTGTTAGTTGTTGAC	23	490–512	1914	[57]
	Emnd5-R	AACCACAGACATATCTATATCG	22	2382–2403		
*nad*1	Emnd1-F	GAGTTTGCGTCTCGATGATAGG	22	7386–7407	1126	[57]
	Emnd1-R	TCCCCAAAACCCACATTCTAC	21	8491–8511		
*cox*1	Emco1-F	AGGTTTGACTTTCTCTTTGGTT	22	9072–9093	1801	[57]
	Emco1-R	GGCAAATAAACCTAAACAACC	21	10,852–10,872		
*cob*	Emcob-F	GTTTAAACTGGTAGATTGTGGTTC	24	3055–3078	1323	[25]
	Emcob-R	CTCCACAGTAGAAATCACCATCA	23	4377–4355		
*cox*3	Emco3-F	TAAATATAGAACGAAAGTAAAT	22	2180–2201	1012	This study
	Emco3-R	TACAAACCCACTACTTCAATAA	22	3191–3170		
*nad*4L	Emnd4L-F	ATTGACTTATTTAGGTGGGTGT	22	4073–4094	1364	This study
	Emnd4L-R	TAGTCGGAAATGAACATAACCT	22	5436–5415		
*nad*3	Emnd3-F	GATTATGGGGAGTCTGAAAGGG	22	8088–8109	1179	This study
	Emnd3-R	TAAACCAACAAAACCAGACCAT	22	9266–9245		
*nad*6	Emnd6-F	GGTGGGGAAAATCAGGCGGTTG	22	12,728–12,749	959	This study
	Emnd6-R	AACACCAAAAAACCAAACACTA	22	13,686–13,665		
*nad*4	ND4Em-F	TACTGTGGAGATTATTATTAGG	22	4368–4389	1519	This study
	ND4Em-R	CAAAATCATTCACAATAACCAT	22	5886–5865		
*atp*6	ATPEm-F	AGTGATGGTTTAATGAGGTGTT	22	5668–5689	799	This study
	ATPEm-R	ACAACACAAAAAACAAAACAAC	22	6466–6445		
*nad*2	ND2Em-F	TTTTCGATTGATCATTAGGTTG	22	6363–6384	1179	This study
	ND2Em-R	CAAACTAAGCAATAAGCCAAAA	22	7541–7520		
*cox*2	Co2Em-F	CAGTAGACTTTTTTGTTGAATG	22	11,798–11,819	1489	This study
	Co2Em-R	AAACCTCCAACAACATAAATCT	22	13,286–13,265		

*atp*6 ATP synthase F0 subunit 6 gene, *cox*1–*cox*3 cytochrome *c* oxidase subunit 1, 2, 3 genes, *cob* cytochrome *b* gene, *nad*1–*nad*6, *nad*4L NADH dehydrogenase subunit 1, 2, 3, 4, 5, 6, 4L genes

**Table 3:** Primers for PCR amplification for nuclear genes of *Echinococcus multilocularis* with positions based on reference sequences (FN567985, FN568375)

Gene	Primer name	Primer sequence (5′–3′)	Length	Position	Size (bp)	References
*elp*-exons VII and VIII	Elp7/8-F	GACTAAGTTTCACTAAGCTCTA	22	2566–2587	636	[25]
	Elp7/8-R	GCTTCCAAGCTAAATCTGCGTAC	23	3201–3179		
*elp*-exon IX	Elp9-F	TTGCATCAATGAATCGGTATTA	22	3934–3955	325	[25]
	Elp9-R	CCGCTCTCGAATACTTTAATGGC	23	4258–4236		
pepck	Pepck-F	AGCAAGGCCGAAGCCGATAAGA	22	6–27	1606	This study
	Pepck-R	GCAAAACGACCATGACTATCCA	22	1611–1590		
*ef1-*α	Ef1a-F	CTGGTAAATCGACTTCCACGGG	22	5–26	1320	This study
	Ef1a-R	GCCTTGGCTTCCTTATCCTTGA	22	1324–1303		

*ef1-*α elongation factor-1 alpha gene, *elp* ezrin–radixin–moesin (ERM)-like protein, *pepck* Phosphoenolpyruvate carboxykinase

Each primer pair was used in a separate polymerase chain reaction (PCR) reaction. The reaction contained 5 µl 5 × prime STAR GXL Buffer (Mg^2+^ plus) and 2 µl of deoxynucleoside triphosphate (dNTP) mixture (2.5 mmol/L each) in a final concentration of 100 µmol/L, 0.5 µl Prime STAR GXL DNA Polymerase (0.625 U/25 µl) (Prime STAR^®^ GXL DNA Polymerase, Cat. ≠ R050A, TAKARA Bio Inc., Japan), 0.5 µl (10 pmol) of each primer with a final concentration of 0.2 μmol/L each, 0.5 μl of 20–50 ng of purified genomic DNA, and nuclease-free water up to the final volume of 25 µl.

A Touchdown PCR with the following programs: initial denaturation at 98 ℃ for 3 min, 10 cycles of 95 ℃ for 30 s, 60 °C for 30 s (sequential decrease of 0.5 ℃ in each cycle), and 72 ℃ for 2 min; then 25 cycles of 95 ℃ for 30 s, 55 ℃ for 30 s, and 72 ℃ for 2 min; and a final elongation step at 72 ℃ for 10 min modified after Laurimäe et al. [31] was used.

Gel electrophoresis (1.5%) made from 1 × TAE (40 mmol/L Tris–acetate, 2 mmol/L EDTA, pH 8.5) stained with TS-GelRed (Cat. TSJ003, TSINGKE, China) was used to visualize 5 μl of the amplified PCR products. The amplicons were sequenced afterwards (Beijing TSINGKE Biotechnology Co., Ltd., Beijing, China) using the same set of primers.

### Molecular analysis

Nucleotides were inspected for errors in UGENE program [32]. The species of each isolate was confirmed through NCBI BLAST search. Sequence comparison was performed separately for each mitochondrial and nuclear gene and for the near-complete mitochondrial genome. Phylogenetic and evolutionary analyses were carried out in MEGA v7 [33]. Furthermore, a Bayesian phylogenetic tree was constructed using MrBayes v. 3.1.2 [34] with a Markov Chain Monte Carlo sampling method to determine the posterior distributions of parameters using a chain length of 4,000,000. The parameters were logged every 1000 generations, with the first 25% discarded as 'burn-in'. TreeView v. 1.6.6 (University of Glasgow, Glasgow, UK) was used to visualize the resulting trees.

Phylogenetic analysis was repeated for *nad*1, *cob*, *nad*2, *nad*5, and the near-complete mitochondrial genome datasets. *Taenia solium* (accession number: AB086256 and NC_004022) was used as the outgroup. Computation of the population diversity and neutrality indices, such as Tajima's D [35] and Fu's Fs [36], was achieved by generating haplotype files in DnaSP v6 [37] and exporting them to the population genetics program Arlequin v35.2.2 [38]. *Taenia solium* (NC_004022) was used as an outgroup for the estimation of Fu’s indices. For the sequence data of the *cob* and *nad*2 genes, each of which has more than five haplotypes, diversity and neutrality indices were calculated. Finally, PopART was used to visualize the haplotype and networks [39].

## Results

### Cyst characteristics, PCR amplification and sequencing

**Fig. 1:**
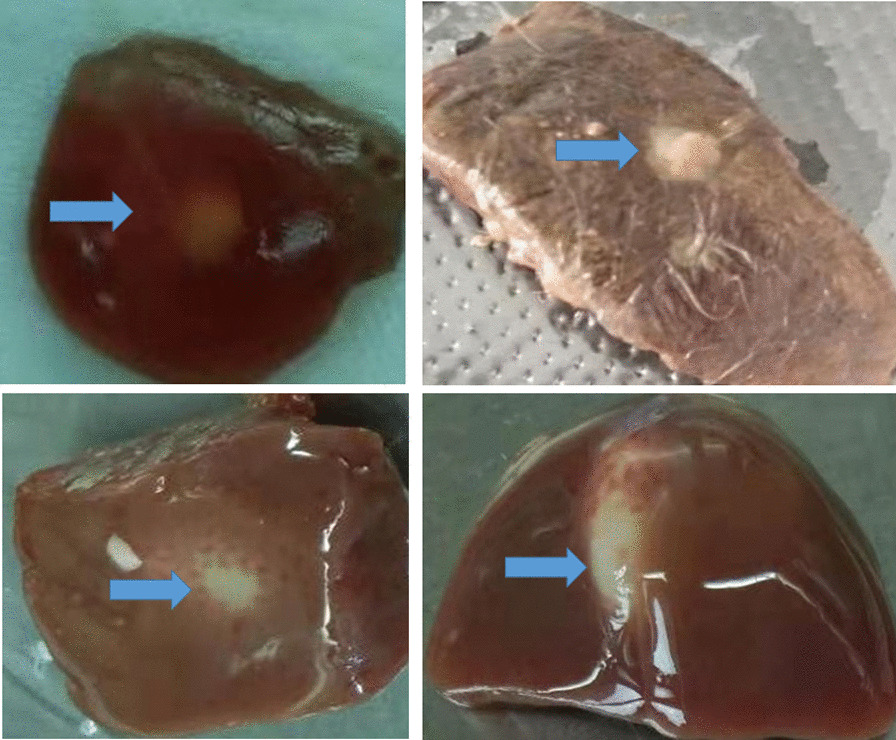
Macroscopic images of lesions confirmed by molecular analysis as alveolar cysts. Blue arrow shows the small white spots

The suspected specimens were distributed as follows: 3 from Jingyuan and one each from Pingchuan, Minqin, Tianzhu, Gaotai, Hezheng, and Jiuquan. The cysts' morphological characteristics differed from those of classical *Echinococcus* cysts. They were all located in the liver, small in size, and without cystic fluid (Fig. 1).

As shown in Table 4, employing the near-complete *mt* genes specific to *E. multilocularis*, at least three primers and as many as nine primers were successfully amplified and sequenced for a single sample. With *nad*4 and *nad*2, all nine specimens were successfully amplified and sequenced, but not with ATP synthase F0 subunit 6 gene (*atp*6) and cytochrome c oxidase subunit 1 (*cox*1) genes. Only one specimen was amplified using *cox*2, nonetheless sequencing was unsuccessful due to poor DNA quality.

**Table 4:** Summary of PCR results using near-complete mitochondrial genome

Sample ID	*nad*5	*cox*3	*cob*	*nad*4L	*nad*4	*atp*6	*nad*2	*nad*1	*nad*3	*cox*1	*cox*2	*nad*6
Hezheng	−	−	−	−	+	−	+	+	−	−	−	−
Tianzhu	−	−	−	−	+	−	+	−	−	−	−	−
Pingchuan	+	+	+	+	+	−	+	+	+	−	−	+
Jingyuan2	−	+	−	−	+	−	+	−	−	−	−	+
Jingyuan3	+	+	+	−	+	−	+	+	+	−	−	+
Jiuquan	+	+	+	−	+	−	+	+	−	−	−	−
Jingyuan5	+	+	+	+	+	−	+	+	+	−	−	−
Gaotai	+	+	+	−	+	−	+	+	+	−	−	+
Minqin	+	+	+	+	+	−	+	+	+	−	+^a^	+

+ successfully amplified and sequenced; − not successfully amplified

*atp*6 ATP synthase F0 subunit 6 gene, *cox*1–*cox*3 cytochrome *c* oxidase subunit 1, 2, 3 genes, *cob* cytochrome *b* gene, *nad*1–*nad*6, *nad*4L NADH dehydrogenase subunit 1, 2, 3, 4, 5, 6, 4L genes: ^a^amplified but not sequenced (due to low concentration)

The *nad*1 (894 bp), cytochrome b (*cob*) gene (1068 bp), *nad*2 gene (874 bp), and *nad*5 gene (1575 bp) sequences' BLAST results showed 98–100% similarity to the *E. multilocularis* reference sequence (AB018440, Japan; MH259779–MH259785, China; MH259784, Qinghai, China). Similarly, the concatenation of nine *mt* genes was > 99.3% similar to the *E*. *multilocularis* reference sequence (GenBank ID: AB018440, Japan). Likewise, all three nuclear genes; *pepck* (1545 bp), *elp*-exons VII and VIII (566 bp), and *elp*-exon IX (256 bp), were only successfully sequenced for a specimen and confirmed the infection was actually caused by *E. multilocularis* with 98.42–99.93% similarity to reference sequences (GenBank FN567985, Hokkaido, Japan; AJ012663, Germany). All resulting nucleotide sequences have been deposited in GenBank under the following accession numbers: OR047885–OR047893 (*nad*1), OR047894–OR047899 (*cob*), OR047900–OR047905 (*nad*5), and OR047906–OR047914 (*nad*4).

### Phylogenetic analysis

**Fig. 2:**
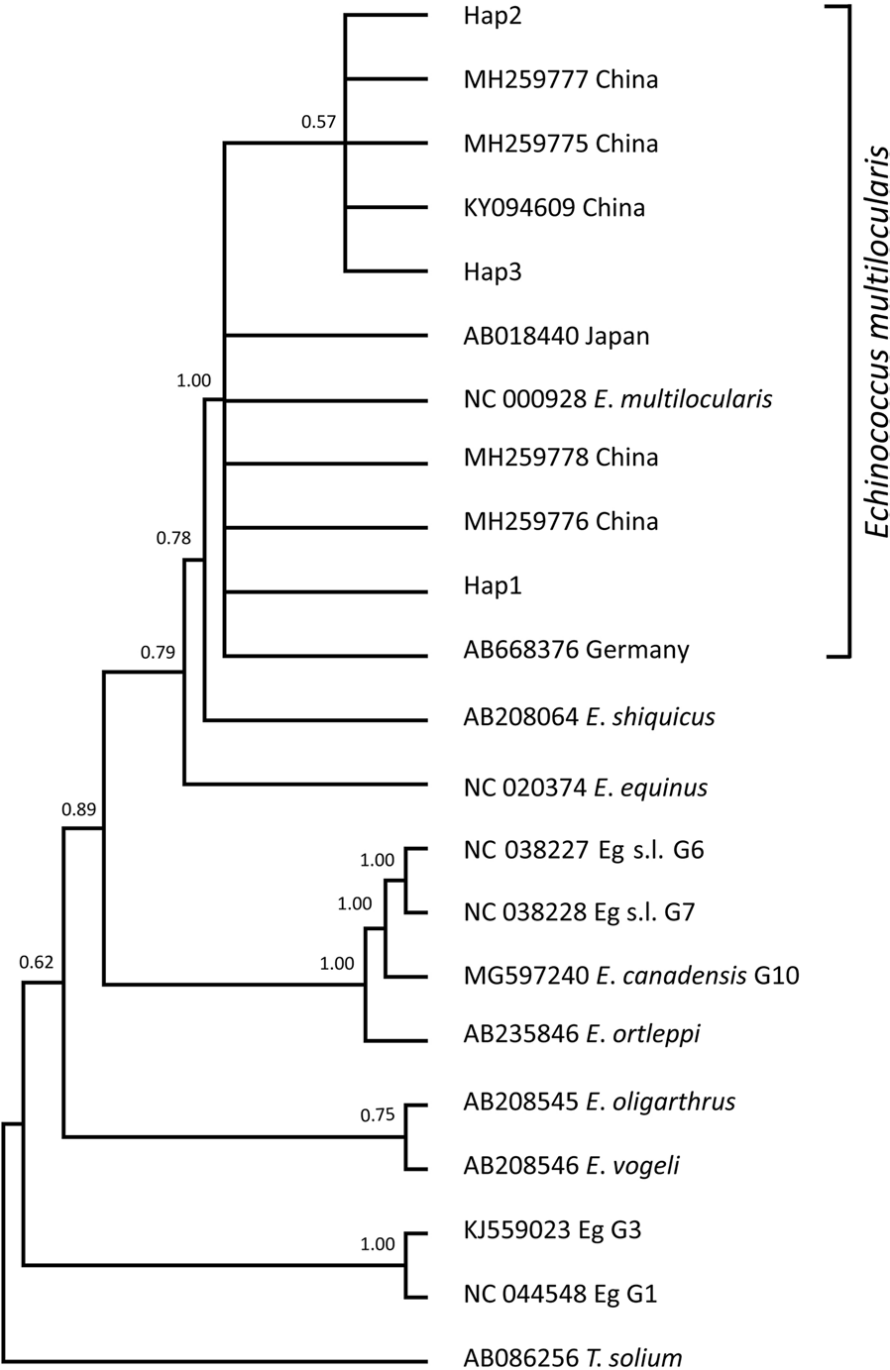
Bayesian phylogeny of the current *Echinococcus multilocularis* isolates inferred from the *nad*1 (894 bp) gene. Posterior probability values are depicted at the nodes. Hap1, Hap2 and Hap3 indicates haplotypes representing isolates from this study. *Taenia solium* (AB086256) was used as the out-group sequence data. Eg G1, Eg G3: *Echinococcus granulosus* genotype 1, 3; Eg s.l.: *Echinococcus granulosus* sensu lato

**Fig. 3:**
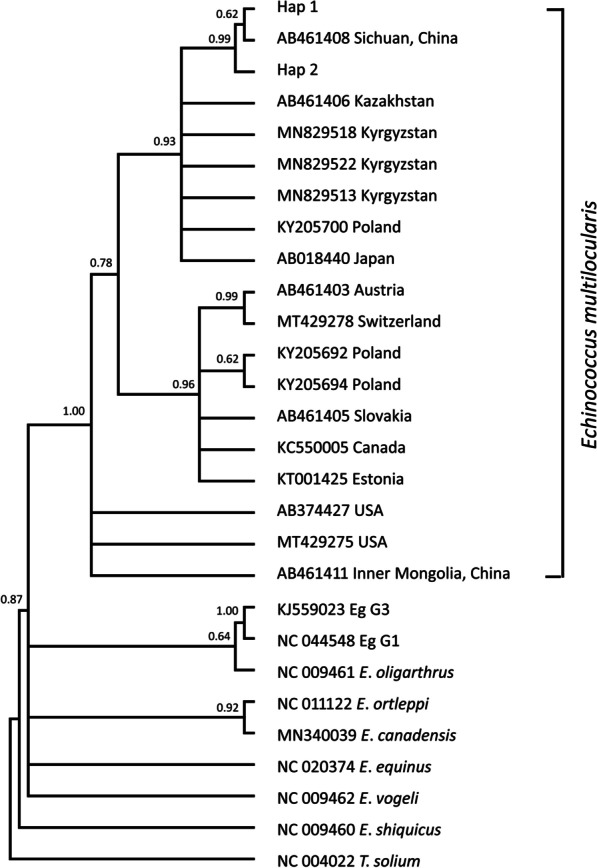
Bayesian phylogeny of the current *Echinococcus multilocularis* isolates inferred from the *nad*2 (874 bp) gene. Posterior probability values are depicted at the nodes. Hap1 and Hap2 indicates haplotypes representing isolates from this study. *Taenia solium* (NC_004022) was used as the out-group sequence data. Eg G1, Eg G3: *Echinococcus granulosus* genotype 1, 3

**Fig. 4:**
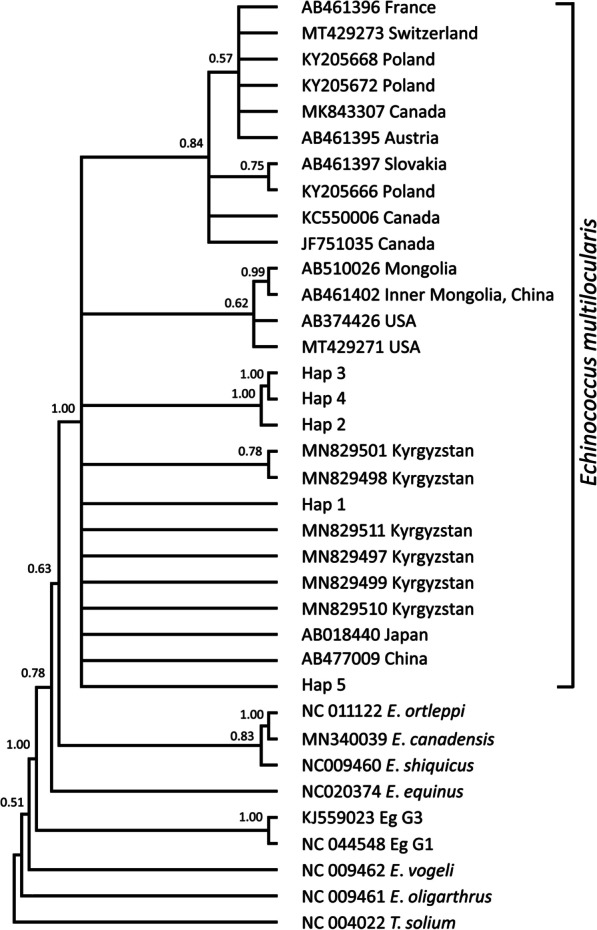
Bayesian phylogeny of the current *Echinococcus multilocularis* isolates inferred from the *cob* (1068 bp) gene. Posterior probability values are depicted at the nodes. Hap1, Hap2, Hap3, Hap4 and Hap5 indicates haplotypes representing isolates from this study. *Taenia solium* (NC_004022) was used as the out-group sequence data. Eg G1, Eg G3: *Echinococcus granulosus* genotype 1, 3

**Fig. 5:**
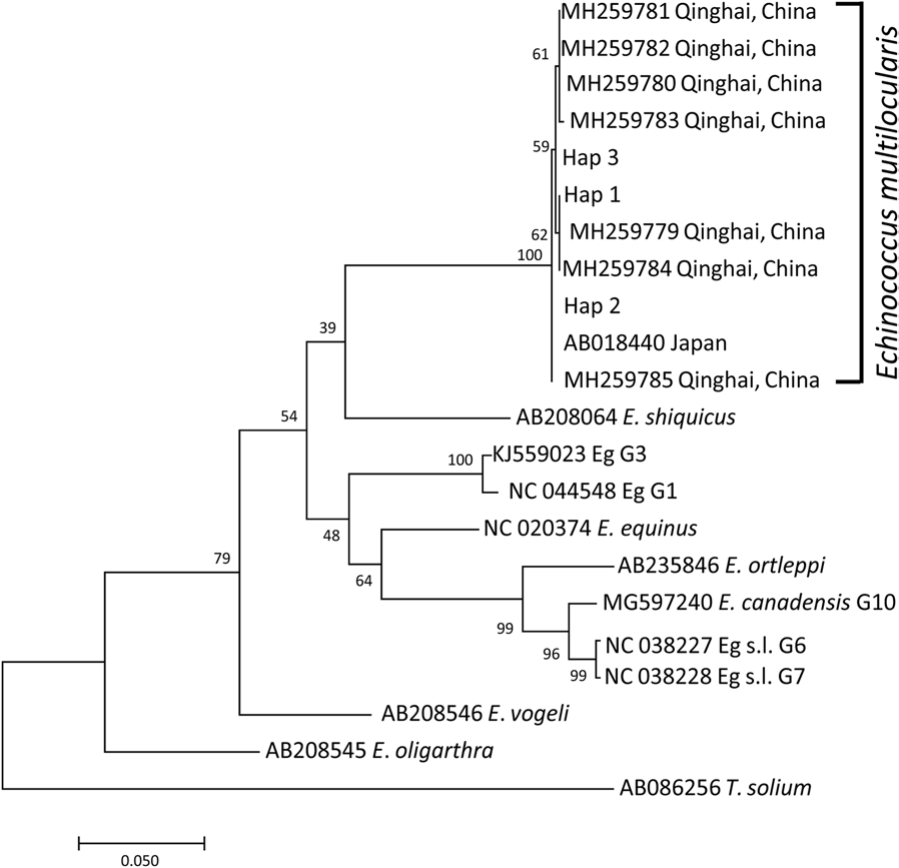
Molecular Phylogenetic analysis by Maximum Likelihood method of the current *Echinococcus multilocularis* isolates inferred from the *nad*5 (1575 bp) gene. Support value greater than 0.39 are displayed to show posterior probability. *Taenia solium* (AB086256) was used as the out-group sequence data. Eg G1, Eg G3: *Echinococcus granulosus* genotype 1, 3; Eg s.l.: *Echinococcus granulosus* sensu lato

**Fig. 6:**
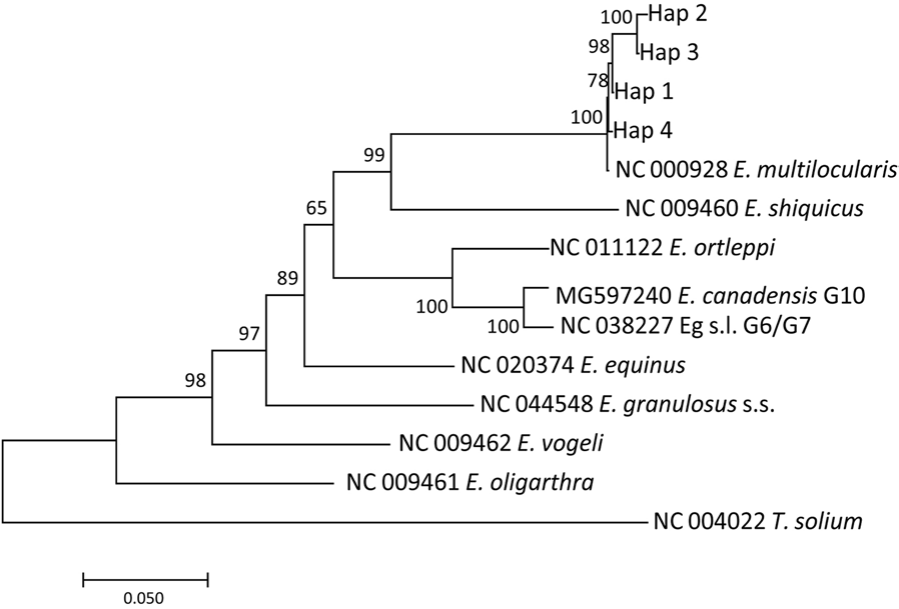
Molecular Phylogenetic analysis by Maximum Likelihood method of the current *Echinococcus multilocularis* isolates inferred from the concatenated sequences (*nad*5, *cox*3, *cob, nad*4, *nad*2, *nad*1, *nad*3, *nad*6; 7005 bp). Support value greater than 0.65 are displayed to show posterior probability. *Taenia solium* (NC_004022) was used as the out-group sequence data. *E*. *granulosus* s.s.: *Echinococcus granulosus* sensu stricto; Eg s.l.: *Echinococcus granulosus* sensu lato

The Bayesian phylogeny of the*nad*1 (894 bp) sequences confirmed the species status of all the isolates as *E. multilocularis*, as they formed a clade with *E. multilocularis* from other hosts and locations (Fig. 2). This was further verified through multiple phylogenetic constructions using *nad*2 (874 bp), *cob* (1068 bp), *nad*5 (1575 bp), and 7005 bp concatenated sequences of 8 genes (*nad*5, *cox*3, *cob, nad*4, *nad*2, *nad*1, *nad*3, *nad*6) (Figs. 3, 4, 5, 6).

### Network analysis

**Fig. 7:**
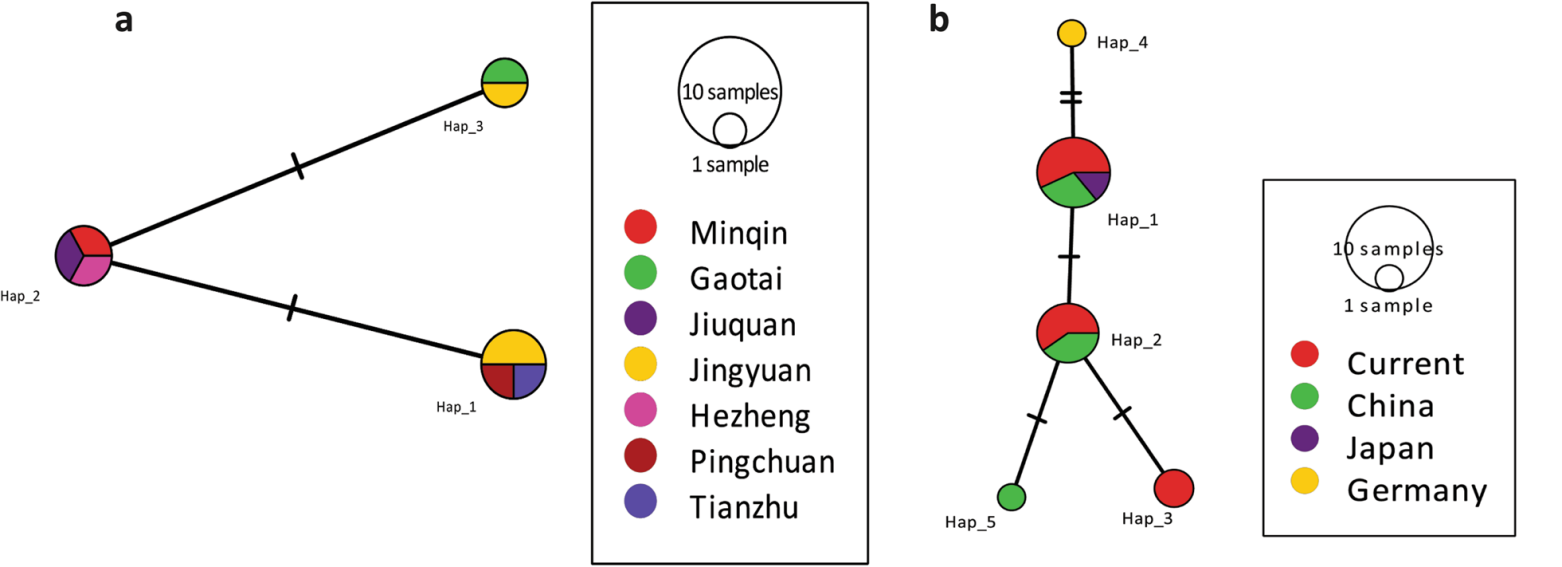
Median-joining networks of the *nad*1 mitochondrial genes of the current isolates (**a**) and 16 sequences consisting of *Echinococcus multilocularis* representative isolates from the world, *nad*1 mitochondrial gene (**b**). Reference sequences, GenBank: KY094609, MH259775–MH259778, AB018440, AB668376. Circle sizes are proportional to the haplotype frequencies. Hatch marks represent the number of mutations

**Fig. 8:**
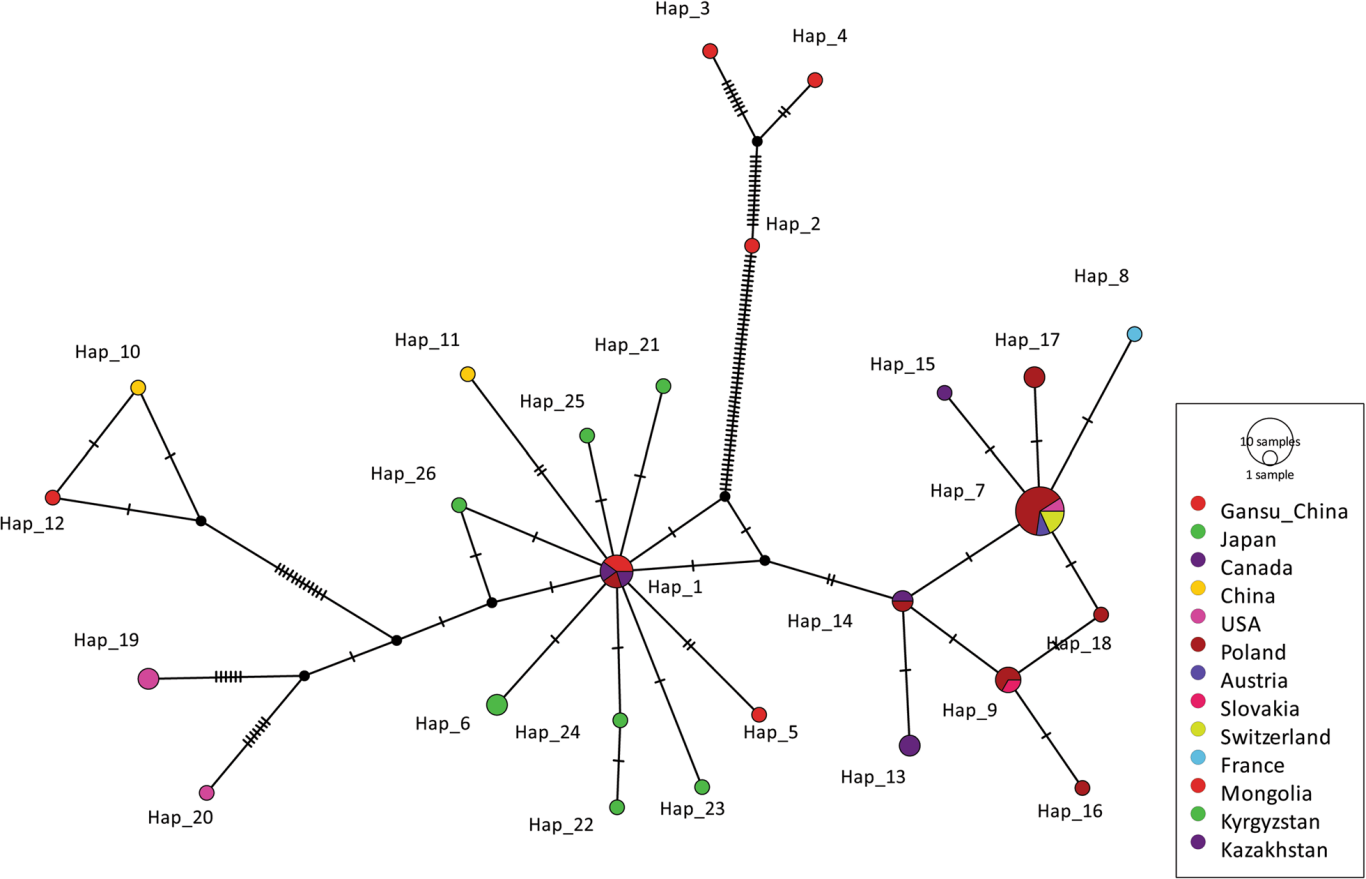
Median-joining network of the *cob* mitochondrial genes of 47 *Echinococcus multilocularis* representative sequences from the world, including the current isolates (Gansu_China); Reference sequences, GenBank: AB018440, AB374426, AB461395–AB461400, AB461402, AB477009, AB510026, JF751035, KC550003, KC550006, KY205662–KY205676, LC380926, MK843307, MN829477–MN829499, MN829501, MN829510–MN829511, and MT429271–MT429274. Circle sizes are proportional to the haplotype frequencies. Hatch marks represent the number of mutations. The small black dots denote median vectors (i.e. hypothetical/unsampled haplotypes)

The median-joining network constructed with the *nad*1 (894 bp) sequences showed two singleton variable sites and two parsimony informative sites (Fig. 7a), and using reference sequences from China (KY094609, MH259775–MH259778), Japan (AB018440), and Germany (AB668376) revealed five mutations (Fig. 7b). Similarly, the median-joining network constructed with the *cob* gene (1068 bp) showed more mutation sites (27 singletons and 93 parsimony-informative sites). Haplotype Hap_7 was common (23.4%, 11/47) in the population and was mainly composed of European (*n* = 10) isolates. A variety of haplotypes appeared as a radial network through various mutational steps (Fig. 8).

**Fig. 9:**
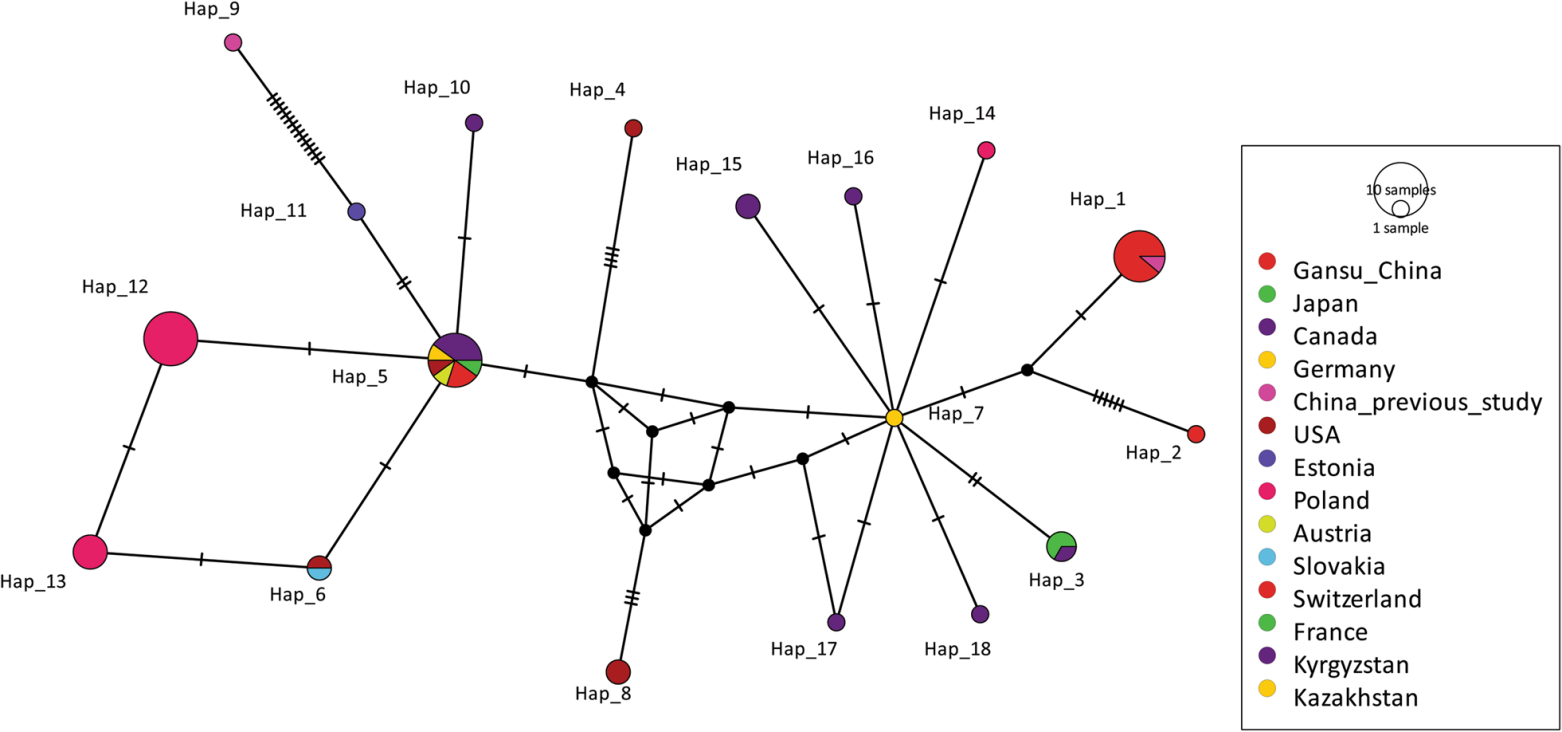
Median-joining network of the *nad*2 mitochondrial genes of 52 *Echinococcus multilocularis* representative sequences from the world, including the current isolates (Gansu_China); Reference sequences, GenBank: AB018440, AB374427, AB461403–AB461409, AB461411, JF751036, KC550005, KC550008, KT001425, KY205692–KY205706, LC380930, MK609520, MN829513, MN829518, MN829522, MN829524, MN829525, MT250265–MT250267, MT429275–MT429278. Circle sizes are proportional to the haplotype frequencies. Hatch marks represent the number of mutations. The small black dots denote median vectors (i.e. hypothetical/unsampled haplotypes)

**Fig. 10:**
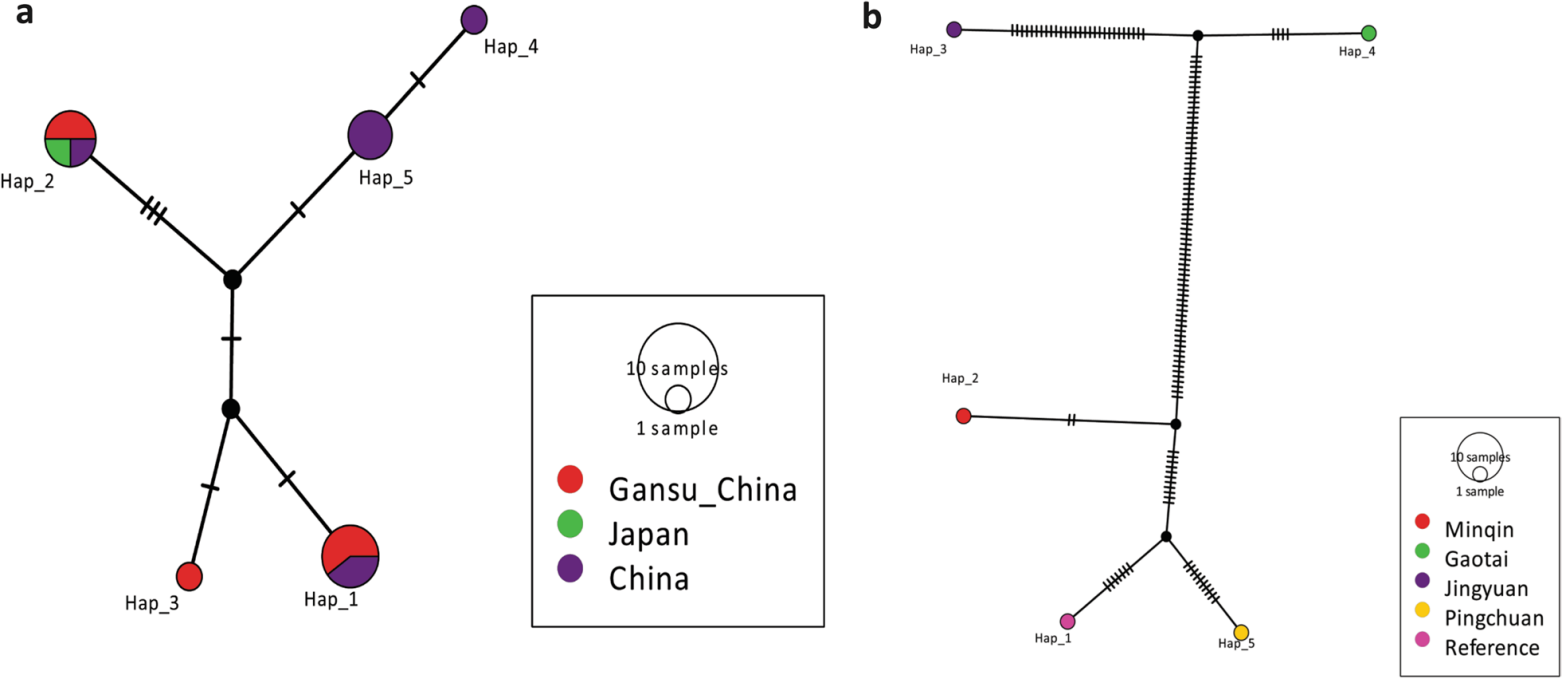
Median-joining network of the *nad*5 mitochondrial genes of 14 *Echinococcus multilocularis* representative sequences from the world, including the current isolates (Gansu_China); Reference sequences, GenBank: AB018440, MH259779–MH259785 (**a**) and the concatenated sequences (*nad*5, *cox*3, *cob, nad*4, *nad*2, *nad*1, *nad*3, *nad*6; 7005 bp) mitochondrial genes of 5 *Echinococcus multilocularis* sequences, including the current isolates (Minqin, Gaotai, Jingyuan, Pingchuan); Reference sequences, GenBank: NC_000928 (**b**). Circle sizes are proportional to the haplotype frequencies. Hatch marks represent the number of mutations. The small black dots denote median vectors (i.e., hypothetical/unsampled haplotypes)

Moreover, in the median-joining network constructed with the *nad*2 gene (874 bp), haplotypes (Hap_5 and Hap_12) were common, each making up 19.2% (10/52) of the population (Fig. 9). Between the present *E. multilocularis* isolates and reference sequences, 25 singletons and 16 parsimony-informative sites were found. Likewise, the median-joining network (Fig. 10a) generated on the *nad*5 gene (1575 bp) showed eight mutations (two singletons and six parsimony-informative sites), and some of the present isolates share the same haplotype with previously described Chinese and Japanese isolates. Finally, the concatenated sequences of 7005 bp (*nad*5, *cox*3, *cob, nad*4, *nad*2, *nad*1, *nad*3, *nad*6) showed 120 mutations (41 singletons and 79 parsimony-informative sites) (Fig. 10b).

### Population and diversity indices

There were 26 distinct haplotypes of the *cob* gene (1068 bp). The haplotype diversity index for the population (*n* = 47) was 0.932, the nucleotide diversity index was 0.01267 while Tajima’s D (− 1.89298) had a statistically significant *P*-value (*P* < 0.05), and Fu’s Fs (− 0.98675) were not statistically significant (*P* > 0.10) when *T. solium* (NC_004022) was used as an outgroup. Similarly, there were also 18 distinct haplotypes of the *nad*2 gene (874 bp). The haplotype diversity index for the population (*n* = 52) was 0.896, the nucleotide diversity index was 0.00633, Tajima’s D (− 1.76799) had a statistically significant *P*-value (*P* < 0.05), and Fu’s Fs (− 2.78572) were not statistically significant (*P* > 0.10) when *T. solium* (NC_004022) was used as an outgroup.

## Discussion

In some parts of China, the *Echinococcus* species *E. granulosus* sensu stricto (*E*. *granulosus* s.s.), *E. multilocularis*, and *E. shiquicus* coexist [40–42]. Meanwhile, *E*. *granulosus* s.s. was confirmed to be mainly transmitting among synanthropic hosts, such as dogs and livestock, while transmission patterns of *E*. *multilocularis* and *E*. *shiquicus* entail intricate sylvatic cycles involving a variety of animal species [43].

Previous findings have indicated that *E. multilocularis* metacestodes have been found in natural settings in a range of mammalian hosts that are not involved in the transmission cycle, such as horses, domestic and wild pigs, nutria (*Myocastor coypus*), various species of captive monkeys, and others [44, 45]. A study conducted in Japan between 1993 and 1994 showed 0.14% of one million slaughtered pigs and 0.82% of 1100 horses both had *E. multilocularis* lesions [46]. The necropsy of those animals may reveal asymptomatic liver abnormalities (either calcified or deteriorated cysts or fertile cysts). Because it's difficult to distinguish these lesions apart from other, more typical lesions like “white spots” or “dense white foci,” molecular testing might be utilized to track suspected lesions [46]. Similarly, domestic ruminants have reported extensive liver lesions that were mistaken for AE, but PCR revealed that these were unusual growth forms of CE [47], as stated in the Panel on Animal Health and Welfare [48]. Molecular testing is therefore required to confirm liver lesions in these animals, particularly in regions where echinococcosis is endemic [49]. In the meantime, unrelated animal species from outside the parasite's geographic range (such as wallabies and hyraxes) have unintentionally contracted the disease [48]. Additionally, certain nonhuman primates, including monkeys and apes, are extremely vulnerable to AE [50]. In the lab, large herbivore infections invariably failed, resulting in small, calcified liver lesions [51].

In the current finding, based on the near-complete mitochondrial and nuclear gene analysis, sheep were found to be infected by *E. multilocularis*. Inline to this, Zhao et al. [52] have reported transmission of CE and AE in domestic animals in Gannan Tibetan Autonomous Prefecture, China as “113 out of 1021 (11.1%) sheep were found infected with CE, and 3 (0.3%) with AE; and 126 out of 634 (19.9%) yaks were infected with CE, and 2 yaks (0.3%) with AE”. Additionally, previous reports showed that dogs, zokors, and pikas (5%, 1% and 2%, respectively) were all found to have *E. multilocularis* infection in South Gansu Province, China [52], suggesting that a life cycle might continue in the area without the presence of wild canids [14]. These findings might give an insight into the possibilities of transmission of *E. multilocularis* in domestic animals such as sheep in endemic areas.

On the other hand, previous reports showed that experimentally introduced eggs in lambs' lungs had the ability to hatch and develop into *Echinococcus* cysts, suggesting that aerosol transmission, which causes lung cysts, is a potential possibility [53, 54]. The anomalously high numbers of pulmonary CE may also be explained by the cold, continental weather patterns that persist for a significant portion of the year in a particular region. These patterns may allow eggs to survive in dust samples, which would then facilitate transmission to humans [54]. Meanwhile, dogs that consume the excrement of wild canids or other dogs, "graze" on grass, or drink impure surface water in areas that may be polluted with the feces of wild canids are at risk of contracting AE in areas where *E. multilocularis* is endemic [55]. This could explain the transmission pattern of *E*. *multilocularis* in the present study.

Inferred from molecular and phylogenetic data, *E. multilocularis* isolates from several intermediate and final Asian hosts form a well-defined, geographically characteristic clade that is distinctly different from isolates from Europe and North America [25]. As was already mentioned, the three significant *Echinococcus* species are getting established in western China [40–42], so it is vital to consider the possibility of coinfection. This claim is confirmed by Santa et al. [56] from Canada, who discovered that while other potential hosts, including coyotes and foxes, have yet to be documented, coinfections of *E. canadensis* and *E. multilocularis* have only recently been detected in wolves.

The current study's unfortunate limitations are the lack of histopathology of the lesion structure and the maintenance of the samples for further processing. The study also failed to provide a clear explanation of how the transmission might have occurred.

## Conclusions

The cysts did not have the same morphological characteristics as conventional *Echinococcus* cysts. The cysts, which were discovered in the livers of sheep, were small white patches devoid of cystic fluid. The present isolates revealed that the infection in sheep was caused by *E. multilocularis* based on near-complete mitochondrial and nuclear gene analysis, but we were unable to provide a clear explanation for how the transmission could have occurred. In endemic areas where CE and AE are prevalent, it is recommended to take into account that domestic animals, such as sheep, could be infected by *E. multilocularis*. As a result, given the importance of the parasite to public health, there is a need for increased surveillance in the region in particular and in the country in general following the detection of *E. multilocularis* in an uncommon intermediate host, which is known to have the highest zoonotic potential.

### Supplementary Information

**Additional file 1:** **Figure S1. **Geographical administrative division at the municipality level, Gansu Province, China.

## Data Availability

All data generated or analyzed in this paper are provided as Additional file.

## Abbreviations

*atp*6: ATP synthase F0 subunit 6
AE: Alveolar echinococcosis
BLAST: Basic Local Alignment Search Tool
bp: Base pair
CE: Cystic echinococcosis
*cob*: Cytochrome *b*
*cox*1–*cox*3: Cytochrome c oxidase subunit 1, 2, 3
DNA: Deoxyribonucleic acid
*mt*: Mitochondrial
*nad*1–*nad*6, *nad*4L: NADH dehydrogenase subunit 1–6, 4L
NCBI: National Center for Biotechnology Information
PCR: Polymerase chain reaction
