# Supplementary figures and images for: Infection of sheep by Echinococcus multilocularis in Gansu, China: evidence from mitochondrial and nuclear DNA analysis
Source: Infect Dis Poverty. 2023 Aug 10;12:72. doi: 10.1186/s40249-023-01120-0 (PMC10413491; doi:10.1186/s40249-023-01120-0)

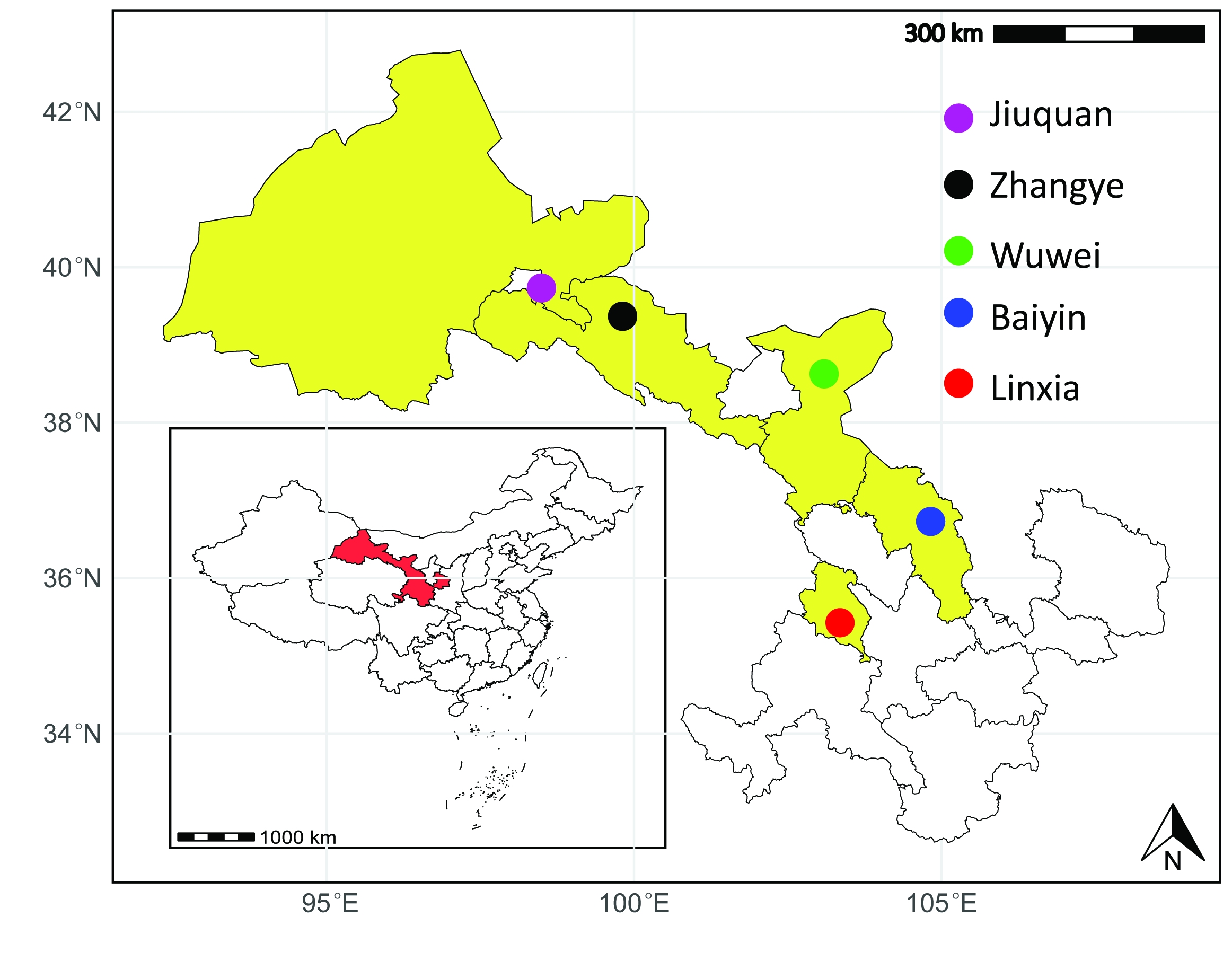

Supplement: Supplementary file 1 — Additional file 1: Figure S1. Geographical administrative division at the municipality level, Gansu Province, China. [file 40249_2023_1120_MOESM1_ESM.tif]
